# Circulating extracellular vesicle-derived MARCKSL1 is a potential diagnostic non-invasive biomarker in metastatic colorectal cancer patients

**DOI:** 10.1038/s41598-023-37008-0

**Published:** 2023-06-20

**Authors:** Wenqing Rong, Shiyun Shao, Yunzhou Pu, Qing Ji, Huirong Zhu

**Affiliations:** Department of Medical Oncology, Shuguang Hospital, Shanghai University of Traditional Chinese Medicine, Shanghai, 201203 China

**Keywords:** Tumour biomarkers, Colorectal cancer, Diagnostic markers

## Abstract

Extracellular vesicle-derived proteins are closely related to colorectal cancer metastasis, and early detection and diagnosis of colorectal cancer metastasis is very important to improve the prognosis. In this study, we evaluated the clinical significance of plasma EV-derived MARCKSL1 in differentiating patients with metastatic and nonmetastatic CRC. This study included 78 patients, including 40 patients with nonmetastatic colorectal cancer, 38 patients with metastatic colorectal cancer, and 15 healthy volunteers. The extracellular vesicles extracted from the participants' plasma were characterized through transmission electron microscopy, nanoparticle tracking analysis and western blotting. MARCKSL1 protein expression in the EVs was detected by ELISA, and the diagnostic efficacy of MARCKSL1 alone or in combination with CA125 and lymphocyte levels was evaluated by receiver operating characteristic curve (ROC) analysis. Pearson's correlation test was performed to detect the correlation between MARCKSL1, CA125, lymphocyte level and clinicopathological characteristics of tumors. The present study demonstrated that the level of circulating EV-derived MARCKSL1 in patients with metastatic colorectal cancer was significantly higher than that in patients with nonmetastatic colorectal cancer and healthy people. Combined with CA125 and lymphocyte levels, the best diagnostic effect was achieved, and the area under the ROC curve was 0.7480. Together, our findings indicated that circulating EV-derived MARCKSL1 could be used as a new potential diagnostic biomarker for metastatic CRC.

## Introduction

Colorectal cancer (CRC) is one of the most common gastrointestinal malignancies in the world. It has a very high morbidity and mortality rate, ranking third in terms of incidence, but second in terms of mortality^1^. An epidemiological survey showed that changes in eating habits and the lack of early screening has led to a continuous increase in the incidence rate and mortality of colorectal cancer in China^2^. Metastasis of colorectal cancer is the main cause of death. Approximately 20% of cancer patients diagnosed for the first time have already experienced metastasis (Phase IV). In addition, approximately 35–45% of stage II and III patients have limited conditions and no metastases, but these patients will recur within 5 years after surgery. In patients with confirmed metastatic colorectal cancer (mCRC), the 3-years survival rate is only 30–35%, and the 5-years survival rate is less than 20%^3^. Therefore, early diagnosis of colorectal cancer metastases is of great significance to improving patient prognosis.

At present, the occurrence of colorectal cancer metastasis is mainly diagnosed by imaging examinations (such as MRI, CT, and PET-CT) and serum tumor markers (such as CEA, CA19-9, and CA50)^3^. Although these examinations can play auxiliary roles in the diagnosis of the disease to a certain extent, there are still many unavoidable shortcomings and limitations. For example, some broad-spectrum tumor markers usually lack clinical accuracy and specificity. There is a possibility of missed diagnosis in the commonly used imaging examinations for some metastatic lesions that are tiny in volume or are present in a more obscure site in the early stage. In addition to imaging and molecular biology, necessary pathological biopsies such as puncture and surgery are highly accurate when tumor metastasis is suspected, but such invasive examinations often increase patients’ pain, and the success rate of puncture and surgery is often affected and limited by the sampling location. Therefore, finding more accurate and convenient metastatic colorectal cancer diagnostic markers is of great significance.

Current studies on circulating diagnostic markers for colorectal cancer mainly include circulating tumor cells (CTCs), circulating tumor DNA (ctDNA) or RNA, protein molecules and extracellular vesicles. These markers have their advantages and disadvantages in diagnosing CRC. For example, the advantage of CTCs is that they can provide cancer-related information at the DNA, RNA and protein levels^4^, and they can reflect tumor detection, treatment monitoring, prediction and individual precision treatment. However, due to their low content in patient serum, short circulating half-life and lack of cancer-specific markers, their detection remains challenging, weakening their value as a diagnostic tool. The half-life of ctDNA is short, so it can be used as a dynamic indicator of tumor progression and a reflection of treatment. Research indicates that it can be used to detect the recurrence of colorectal cancer 2–15 months earlier than CT imaging^5^. However, due to its small proportion in circulating free DNA, it relies on more sensitive detection technology. The advantage of circulating protein is that it is easy to extract and detect, but several protein markers commonly used in the clinic, such as CEA and CA125, are not ideal in distinguishing metastatic colorectal cancer, and their sensitivity and specificity are poor.

Extracellular vesicles (EVs) are small membranous vesicles that are released into the extracellular matrix by cells. Various types of cells release EVs under physiological or pathological conditions^6^. According to the different cell components contained in EVs, cells can produce various physiological activities, such as reshaping the extracellular matrix and transmitting signals and molecules to other cells. As a multifunctional intercell transmission system in the human body, EVs are involved in regulating intercytoplasm communication and play an important role. They can affect tissue homeostasis, immune regulation and the onset and progression of the tumor. The lipid bilayer structure of EVs can protect their contents from being degraded by enzymes in blood circulation, and after sample collection, the extracted EVs can be purified in vitro to improve the concentration of markers in the sample, making the detection more sensitive and the results more stable^7^. In view of the unique structure, function and advantages of EVs, EV-derived markers are gradually attracting the attention of researchers as diagnostic markers for CRC. Among them, EV-derived noncoding RNAs have garnered extensive attention due to their widespread availability and high specificity to CRC^8^, and EV-derived proteins have also drawn attention because of their advantages of stability, long half-life, and direct action on target cells^9^. According to the literature, the expression profiles of EV-derived proteins tend to be significantly different in different stages of cancer, indicating that these proteins are closely related to cancer initiation and progression^10^. Therefore, EV-derived proteins have greater potential as diagnostic markers for CRC. Currently, most existing studies have focused on CRC diagnosis^11,12^, and the identification of EV-derived protein markers for metastatic CRC is still lacking.

As a ubiquitous membrane-associated protein, the functions of myristoylated alanine rich protein kinase C substrate (MARCKS) and MARCKS-like protein 1 (MARCKSL1) are very broad. Through phosphorylation of protein kinase C (PKC) or combination with calcium-dependent calmodulin, MARCKS and MARCKSL1 are transferred to the cytosol and are involved in structural regulation of the actin cytoskeleton, chemotaxis, motility, cell adhesion, phagocytosis and exocytosis, and activation of various signal transduction pathways. As a protein that is closely related to the occurrence and progression of a variety of tumors, MARCKS is mainly expressed in congenital immune cells, which can promote inflammatory immune cell migration and adhesion and promote immune cell secretion of cytokines such as tumor necrosis factor (TNF)^13,14^. MARCKSL1 has been proposed to be a potential differentiator between metastatic and nonmetastatic colorectal cancer by proteomics analysis in relevant clinical studies^15^.

In our previous study, we found that MARCKSL1 was significantly highly expressed in plasma EVs of patients with metastatic colorectal cancer by analyzing the proteomic data of plasma EVs in small samples of healthy people and patients with metastatic and nonmetastatic colorectal cancer. We further conducted a literature search on related studies of MARCKSL1. In the study of Maria Gonzalez-Gonzalez et al.^15^, nucleic acid programmable protein arrays (NAPP Array) were employed to identify aAb in plasma samples from a set of 50 sCRC patients compared to seven healthy donors. MARCKSL1 was found to be a tumor-associated protein (TAA), and its expression in the plasma of sporadic colorectal cancer (sCRC) patients was significantly higher than that of healthy people (the aAbs constituting the sCRC immunome presented mean FC values = 2.4 > 1.5, with more than 88% of sCRC patients showing FC values > 1), but it was not good at distinguishing metastatic and nonmetastatic colorectal cancer.

Based on this, the purpose of this study was to determine whether EV-derived MARCKSL1 can distinguish metastatic colorectal and nonmetastatic colorectal cancer and whether it can be used as an early diagnostic biomarker of metastatic colorectal cancer. Therefore, we studied the relationship between the plasma EV-derived MARCKSL1 level and the tumor stage and clinicopathological markers of CRC in healthy people, patients with nonmetastatic CRC and colorectal cancer to assess its value in the early diagnosis of CRC progression. In addition, we performed a correlation analysis of commonly used tumor markers for colorectal cancer according to the metastatic status of the enrolled patients and selected the tumor marker CA125 as a potential diagnostic marker for inclusion. According to the diagnostic criteria, we evaluated the diagnostic value of MARCKSL1, CA125 and lymphocytes as markers.

## Patients and methods

### Patients and plasma sample preparation

The study group included 93 participants: 40 patients with nonmetastatic colorectal cancer (nm CRC group, 23 women and 17 men), 38 patients with metastatic colorectal cancer (mCRC group, 19 women and 19 men), and 15 healthy volunteers (health group, 7 women and 8 men), all of whom were diagnosed at Shuguang Hospital Affiliated to Shanghai University of Traditional Chinese Medicine between December 2020 to September 2022. By histological tissue examination and imaging, CRC was diagnosed clinically. The plasma samples were collected before surgery or first-line treatment. CRC staging was determined using the tumor node metastatic classification (TNM) staging system created by the Union for International Cancer Control (UICC). In addition, all CRC patients were grouped according to colorectal cancer stage. We divided the mCRC group into subgroups according to the metastatic sites and divided the nmCRC group into subgroups based on the degree of tumor invasion (T factor), lymph node metastases (N factor), distant metastases (M factor), and tumor stage (TNM). Tables 1, 2, 3, 4 illustrates the characteristics of the research groups. All participants signed a written informed consent form, and the project was approved by the Ethics Committee of Shuguang Hospital affiliated to Shanghai University of Traditional Chinese Medicine. The plasma was separated by centrifugation and stored at − 80 °C.

**Table 1:** Characteristics of the enrolled patients.

Parameters	Groups (n = 78)	Number
Gender	Male	36
	Female	42
Age(years, Mean ± SD)	nmCRC	64.15 ± 11.72
	mCRC	66.03 ± 12.09
TNM stage	0-I	6
	II	11
	III	23
	IV	38

Abbreviations: *nmCRC* nonmetastatic colorectal cancer, mCRC metastatic colorectal cancer.

**Table 2:** Characteristics of the enrolled healthy volunteers.

Parameters	Groups (n = 15)	Number
Gender	Male	7
	Female	8
Age (years, Mean ± SD)	62.47 ± 9.94

**Table 3:** Nonmetastatic colorectal cancer (nmCRC) patients' characteristics.

Parameters	Groups (n = 40)	Number
Gender	Male	17
	Female	23
TNM stage	0 + I	6
	II	11
	III	23
Tumor invasion depth (T-factor)	Tis + T1	4
	T2	3
	T3	23
	T4	10
Metastases in lymph nodes (N-factor)	N0	18
	N1	14
	N2	8
Distant metastases (M-factor)	M0	40
	M1	0

Abbreviations: *TNM* stands for tumor (T), nodes (N), and metastases (M).

**Table 4:** Metastatic colorectal cancer (mCRC) patient characteristics.

Parameters	Groups (n = 38)	Number
Gender	Male	19
	Female	19
Sites of metastasis	MD	15
	Li	16
	Lu	3
	BM	1
	AM	2
	PM	1

Abbreviations: *MD* Multiple distant metastases e.g., liver, lung, peritoneum, omentum, mesentery, bone, ovary, adrenal gland; *Li* Liver only, *Lu* Lung only, *BM* Bone marrow only, *AM* Abdominal metastasis only, *PM* peritoneum metastasis only.

### Plasma EVs isolation

According to the manufacturer’s protocol, plasma EVs were extracted using the Hieff^®^ Quick exosome isolation kit (for serum/plasma) (cat.41202ES30, Yeasen Biotechnology, Co., Ltd., Shanghai, China) and cryopreserved at − 80 °C until use. Briefly, a 1 ml plasma sample was centrifuged at 4 °C at 3000× *g* for 10 min. The supernatant was collected and centrifuged at 4 °C at 10,000× *g* for 20 min. Then, 4 ml PBS was added and thoroughly mixed, 1 ml 41,202-A reagent was added to the sample diluted with PBS, vortexed for 1 min and placed in a refrigerator at 4 °C for 2 h. The mixed liquid was centrifuged at 4 °C at 10,000× *g* for 60 min, the supernatant was discarded, and the sediment rich in EVs was collected. Then, 400 μl of PBS was added to the sediment to resuspend the EVs. The suspension was centrifuged at 4 °C at 12,000 × g for 2 min, the sediment was discarded, and the supernatant was retained which contained the purified EVs fraction. BCA assays were used to measure the concentration of total protein, then the EVs preparations were stored at − 80 °C.

### Transmission electron microscopy analysis (TEM)

TEM was conducted at room temperature. Briefly, 5 µl suspension with EVs or PBS without EVs (negative control) were placed on a copper net and incubated for 5 min. At the end of incubation, excess liquid was blotted on one side with blotting paper. A drop of 2% uranium acetate was added to the copper net and incubated at room temperature for 1 min. At the end of incubation, the excess liquid was blotted with blotting paper on one side. The samples were dried at room temperature for approximately 20 min and then observed on the instrument. The instrument we used in the test was a transmission electron microscopy manufactured by FEI, and the instrument model was Tecnai G2 Spirit BioTwin. The acceleration voltage setting was 80 kV.

### Nanoparticle tracking analysis (NTA)

The frozen EV samples were thawed in a 25 °C water bath and placed on ice. PBS (1×) was used to dilute thawed EVs samples for NTA detection. EV samples were diluted with 1× PBS and directly used for NTA detection. The test instrument was a Nanometer Particle Tracking Analyzer manufactured by Particle Metrix, and the instrument model was ZetaView S/N 17-310. The software version for analysis was ZetaView 8.04.02.

### Western blot

EV samples were treated with RIPA (cat. no. P0013C, Beyotime Institute of Biotechnology, Shanghai, China) lysis buffer to obtain EV-proteins. The protein concentration was quantified using a standard BCA protein assay kit (cat. no. P0012S, Beyotime Institute of Biotechnology, Shanghai, China). EV-derived proteins (15 μg) were loaded onto SDS‒PAGE (3% stacking gel, 12% running gel, cat. P0012A, Beyotime Institute of Biotechnology, Shanghai, China) and running in a Mini Protean 2 electrophoresis system (Bio-Rad) then transferred to PVDF membranes (cat. no. IPVH00010, Merck Millipore, MA, USA). PVDF membranes were blocked with 5% BSA at room temperature for 1 h and incubated with primary antibodies CD81(cat. no. 10037, Cell Signaling Technology, MA, USA, 1:1000 dilution) and TSG101(cat. no. ab133586, Abcam, MA, USA, 1:1000 dilution) at 4 °C overnight. After washing with TBST, the membranes were incubated with HRP-conjugated secondary antibodies (cat. no. SA00001-2, Proteintech, Wuhan, China, 1:1000 dilution) at room temperature for 1 h. Again, the membrane was rinsed with 1 × TBST and this was repeated 3 times (5 min per rinse). The proteins transferred onto the PVDF membrane were finally detected by chemiluminescence using the BIO-RAD Chemidoc XRS system with enhanced chemiluminescence reagent (cat. no. WBULS0100, Merck Millipore, MA, USA).

### Determination of EV-derived MARCKSL1, CA125 and lymphocytes level

Plasma EV-derived MARCKSL1 concentrations were measured using an enzyme-linked immunosorbent assay (ELISA) kit (cat. no. ML851570-2, Mlbio, Shanghai, China) according to the manufacturer's instructions. Briefly, EV samples were diluted 1:1 with sample diluent, then 50 µl of Standard, control, or sample were added to each well. 50 μl of the biotin-labeled antibody was added immediately and incubated 1 h at room temperature. The liquid in the wells was shaken off and washed with washing solution 3 times. Subsequently, 80 μl conjugate were added to each well and the plate was incubated at room temperature for 30mi, then each well was washed 3 times again. 50 μl of substrate A and B were added to each well, mixed with gentle shaking, and incubated at 37 °C for 10 min. Eventually, 50 µl of stop solution was added to each well. UV absorption was measured at 450 nm using a plate reader (Multiskan FC, Thermo Fisher Scientific). CA125 was determined by an Abbott I2000 automatic immunochemiluminescence analyzer (Abbott Pharmaceutical Company) following the manufacturer’s protocol. Routine blood-related indices were measured with the Beckman Coulter LH780 hematology analyzer following the manufacturer's protocol.

### Statistical analysis

All data are presented as the mean ± standard deviation (SD). In the preliminary statistical analysis, if the data did not fit a normal distribution (Kolmogorov‒Smirnov test), natural logarithmic transformation was used, and then the normal test was conducted again. If the data transformed followed a normal distribution, parametric statistical methods were used, and we used Student's t test or ANOVA to check if the means of two or more groups were significantly different. Nonparametric statistical tests were used if the transformed date still did not fit the normal distribution. Correlation analysis was performed using Pearson's correlation test. As part of our evaluation, sensitivity, specificity, accuracy, predictability of positive (PPV) and negative (NPV) outcomes, and area under the ROC curve (AUC) were also evaluated. *P* < 0.05 was considered statistically significant. The concentration of MARCKSL1 below the detection limit was set to zero. Analysis of the data was conducted using IBM SPSS Statistics 26.0. Microsoft Office Excel was used to calculate diagnostic parameters. Univariate logistic regression models were established for each risk factor, and multivariate analyses were performed for variables with *P* < 0.05. Bar charts, scatter plots, and other plots were created using the GraphPad Prism 9.2.0 program (GraphPad Software, Inc., version v9.3.1.471).

### Ethics approval and consent to participate

This study was performed in line with the principles of the Declaration of Helsinki. The project was approved by the Ethics Committee of Shuguang Hospital affiliated to Shanghai University of Traditional Chinese Medicine. (Approval number: 2020-852-59-01). Signed consent forms were obtained before participants were enrolled. Informed consent was obtained from all individual participants included in the study.

## Results

### Identification of EVs derived from plasma

**Figure 1:**
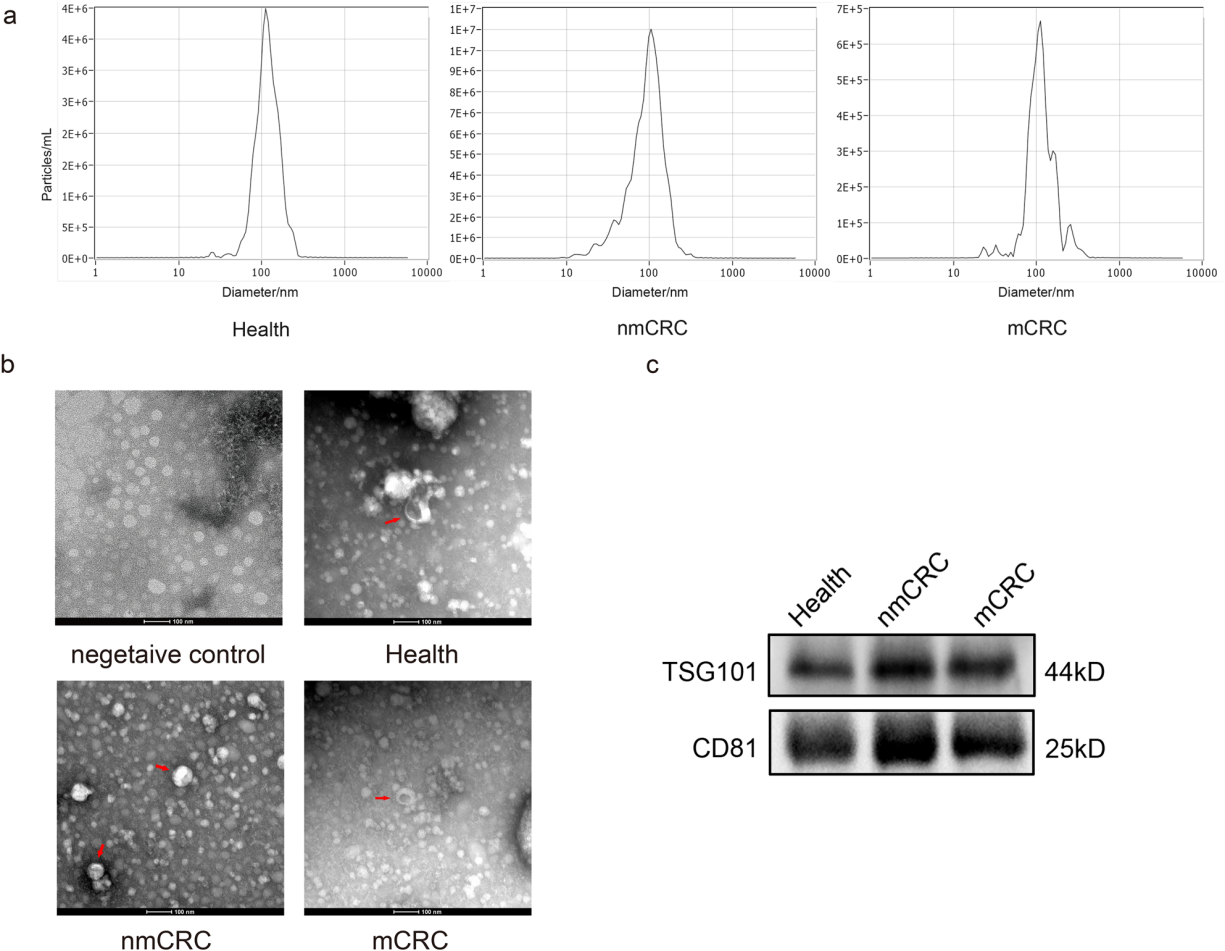
EVs identification. (**a**) NTA data showed that EVs peak sizes were 40–150 nm. (**b**) TEM images showed typical EVs with oval or bowl-shaped microvesicles. (**c**) Western blotting showed that patient plasma EVs were positive for the two EV-derived markers, TSG101 and CD81. Original blots are presented in Supplementary Fig. S1.

Transmission electron microscopy (TEM) was used to confirm plasma-derived EVs. A nanometer particle tracking analyzer (NTA) was used to measure the size and density of EVs, which confirmed the successful isolation of nanoscale vesicles (Fig. 1a). TEM images showed that the extracted EVs were 40–150 nm in diameter, round or cup-shaped and surrounded by a lipid bilayer, while the negative control group did not possess the typical morphology of EVs (Fig. 1b). EVs with typical EV markers CD81 and TSG101 were also characterized by western blotting (Fig. 1c). These results indicated that a wide variety of EVs exist in human plasma, both in the plasma of healthy individuals and cancer patients, and that EVs from different individuals cannot be distinguished solely according to diameter and morphology.

### Plasma levels of MARCKSL1, CA125, lymphocyte, and lymphocyte ratio in CRC patients

**Figure 2:**
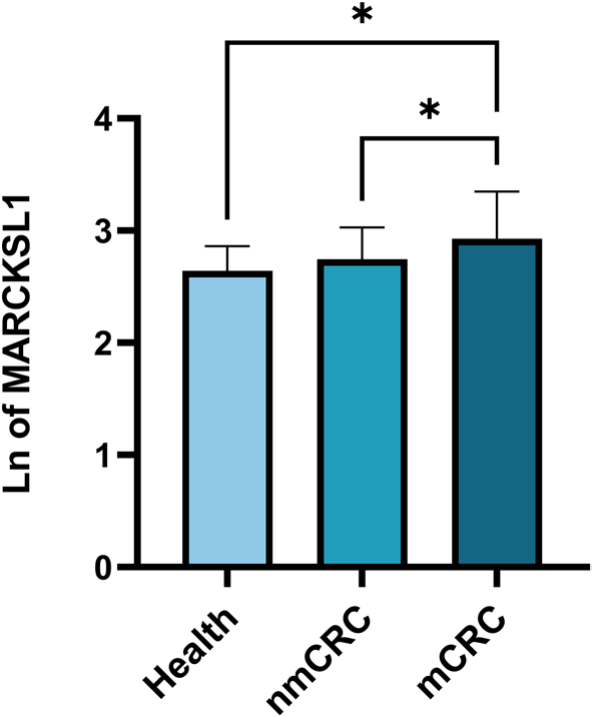
Plasma EV-derived MARCKSL1 concentrations in CRC patients and healthy controls. Data are presented as mean ± SD. Health, healthy volunteers; nmCRC, nonmetastatic colorectal cancer; mCRC, metastatic colorectal cancer; Ln of MARCKSL1, the natural logarithm of the concentration of MARCKSL1. *P < 0.05.

To explore the effect of EV-derived MARCKSL1 and other related indicators in differentiating nmCRC from mCRC, we compared the MARCKSL1, CA125, lymphocyte, and lymphocyte ratios of the patients. Compared with nonmetastatic CRC patients (*P* = 0.0485) and healthy people (*P* = 0.0184), the plasma EV-derived MARCKSL1 level in mCRC patients was significantly elevated. The level of plasma EV-derived MARCKSL1 in nonmetastatic CRC patients was also higher than that in healthy subjects, but no significant difference was found (*P* > 0.05) (Fig. 2). Therefore, we considered MARCKSL1 as a potential marker to distinguish mCRC from nmCRC and performed further analysis.

**Figure 3:**
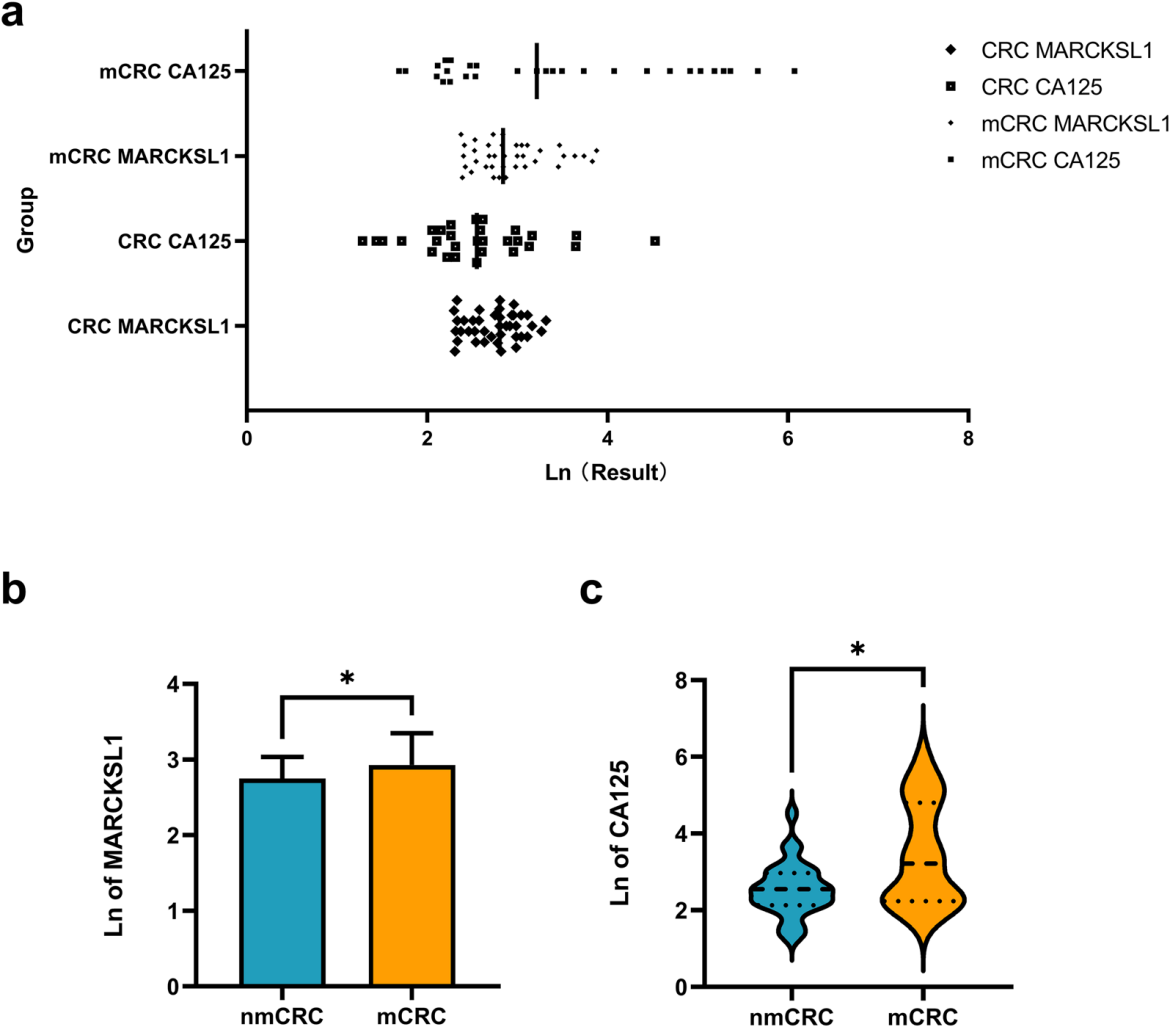
Concentrations of plasma EV-derived MARCKSL1 and plasma CA125 in patients with mCRC in comparison to patients with nmCRC. (**a**)–(**c**) the level of EVs MARCKSL1 (*P* = 0.0278) and CA125 (*P* = 0.0296) in plasma of mCRC patients was much higher than that of nmCRC patients. *P < 0.05. Data are presented as mean ± SD or median (interquartile). nmCRC, nonmetastatic colorectal cancer; mCRC, metastatic colorectal cancer; Ln of concentration, the natural logarithm of the concentration.

**Figure 4:**
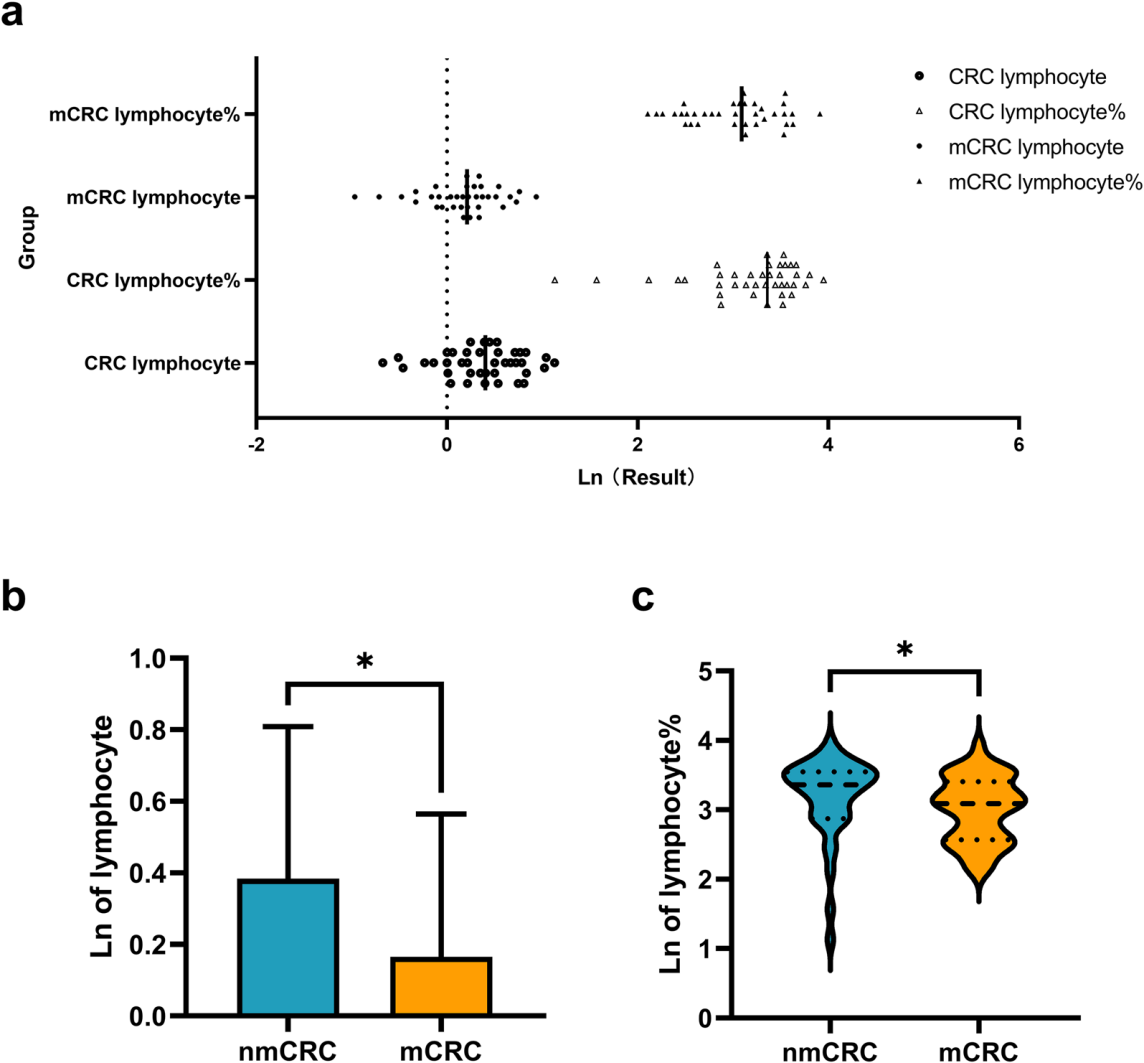
Plasma concentration of lymphocyte and lymphocyte ratio in patients with mCRC in comparison to patients with nmCRC. (**a**)–(**c**) The lymphocyte (*P* = 0.0302) and lymphocyte ratio (*P* = 0.0354) levels decreased in mCRC patients. *P < 0.05. Data are presented as mean ± SD or median (interquartile). nmCRC, nonmetastatic colorectal cancer; mCRC, metastatic colorectal cancer; Ln of concentration, the natural logarithm of the concentration.

Table 5 shows the natural logarithm of the concentrations of plasma EV-derived MARCKSL1 and plasma CA125, lymphocytes and lymphocyte ratios. The expression of all indices in mCRC patients was significantly different from that in nmCRC patients. Specifically, the levels of EV-derived MARCKSL1 (*P* = 0.0278) and CA125 (*P* = 0.0296) in the plasma of mCRC patients were much higher than those in nmCRC patients. The lymphocyte (*P* = 0.0302) and lymphocyte ratio (*P* = 0.0354) levels decreased in mCRC patients (Table 5, Figs. 3, 4).

**Table 5:** The natural logarithm of the plasma concentrations of EV-derived MARCKSL1 and CA125, lymphocyte and lymphocyte ratio in patients with mCRC in comparison to patients with nmCRC.

		MARCKSL1	lymphocyte		CA125	lymphocyte ratio
nmCRC group (n = 40)	Mean ± SD	2.747 ± 0.285	0.3744 ± 0.419	Minimum	1.284	1.131
				Median	2.550	3.360
				Maximum	4.527	3.949
mCRC group (n = 38)	Mean ± SD	2.929 ± 0.414	0.1650 ± 0.394	Minimum	1.686	2.104
				Median	3.215	3.091
				Maximum	6.071	3.912
*P* value	*t* test	0.0278	0.0302	Mann–Whitney test	0.0296	0.0354

Abbreviations: *nmCRC* nonmetastatic colorectal cancer, *mCRC* metastatic colorectal cancer.

### Relationship between plasma levels of MARCKSL1, CA125, lymphocyte, lymphocyte ratio and clinicopathological features in CRC patients

To further analyze the correlation between the tested indices and clinicopathological features, subgroup analysis was performed on all enrolled CRC patients on the basis of metastatic status, TNM stage and metastatic sites.

**Figure 5:**
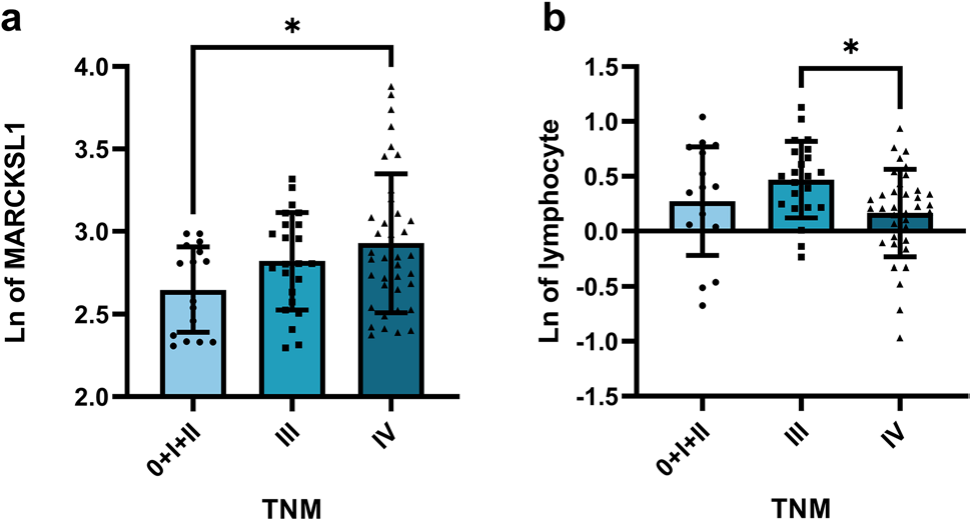
Plasma concentration of EV-derived MARCKSL1 and lymphocyte in CRC patients according to TNM classification. (**a**) The level of MARCKSL1 of stage IV was the highest, the difference between stage 0 + I + II and stage IV patients was significant (*P* = 0.0225). (**b**) The level of lymphocyte of stage IV was the lowest, statistical difference was observed in stage III and IV (*P* = 0.0204). *P < 0.05. Data are presented as mean ± SD. nmCRC, nonmetastatic colorectal cancer; mCRC, metastatic colorectal cancer; Ln of MARCKSL1, the natural logarithm of the MARCKSL1.

After the normality test of all indices, ANOVA was applied to the levels of MARCKSL1 and lymphocytes, and the Kruskal‒Wallis test was used to compare CA125 and the lymphocyte ratio. According to the TNM stage of CRC patients, the levels of MARCKSL1 and CA125 were the highest in stage IV patients, the expression of MARCKSL1 showed a gradient increasing trend with increasing stage, and there was a significant difference between stage 0 + I + II and stage IV patients (*P* = 0.0225; Table 6, Fig. 5a). Lymphocytes had the lowest level in stage IV patients and had a significant difference in distinguishing stage III and stage IV patients (*P* = 0.0204; Table 6, Fig. 5b). The same expression trend could be observed in the lymphocyte ratio, but the difference was not significant (*P* > 0.05).

**Table 6:** Correlation between tested indexes and clinicopathological features.

			MARCKSL1	lymphocyte		CA125	Lymphocyte ratio
TNM (Tumor stage)	0 + 1 + 2 (n = 17)	Mean ± SD	2.627 ± 0.242	0.273 ± 0.480	Minimum	1.284	1.131
					Median	2.754	3.357
					Maximum	4.527	3.802
	3 (n = 23)		2.820 ± 0.287	0.470 ± 0.341	Minimum	1.435	2.116
					Median	2.313	3.369
					Maximum	3.645	3.949
	4 (n = 38)		2.929 ± 0.414	0.165 ± 0.394	Minimum	1.686	2.104
					Median	3.215	3.091
					Maximum	6.071	3.912
*P* value	ANOVA test		0.0294	0.0274	Kruskal–Wallis test	0.0584	0.0755
Distant Metastases	M0 (n = 40)	Mean ± SD	2.747 ± 0.285	0.384 ± 0.419	Minimum	1.284	1.131
					Median	2.550	3.360
					Maximum	4.527	3.949
	M1 (n = 38)		2.929 ± 0.414	0.165 ± 0.394	Minimum	1.686	2.104
					Median	3.215	3.091
					Maximum	6.071	3.912
*P* value	*t* test		0.0278	0.0246	Mann–Whitney test	0.0296	0.0354
Sites of metastases	NM (n = 40)	Mean ± SD	2.747 ± 0.285	0.384 ± 0.419	Minimum	1.284	1.131
					Median	2.550	3.519
					Maximum	4.527	3.949
	MD (n = 15)		2.962 ± 0.433	0.044 ± 0.421	Minimum	1.758	2.104
					Median	3.904	2.779
					Maximum	6.071	3.535
	Li (n = 16)		2.879 ± 0.358	0.336 ± 0.244	Minimum	1.686	2.460
					Median	2.504	3.131
					Maximum	4.916	3.912
	SM (n = 7)		2.974 ± 0.476	0.039 ± 0.464	Minimum	2.251	2.262
					Median	3.318	3.116
					Maximum	5.287	3.561
*P* value	ANOVA test		0.1505	0.0239	Kruskal–Wallis test	0.0195	0.0879

Abbreviations: *NM* Non-metastases, *MD* Multiple distant metastases e.g., liver, lung, peritoneum, omentum, mesentery, bone, ovary, adrenal gland, *Li* Liver only, *SM* Single metastasis in other parts except liver e.g., lung, bone, Abdominal, peritoneum.

**Figure 6:**
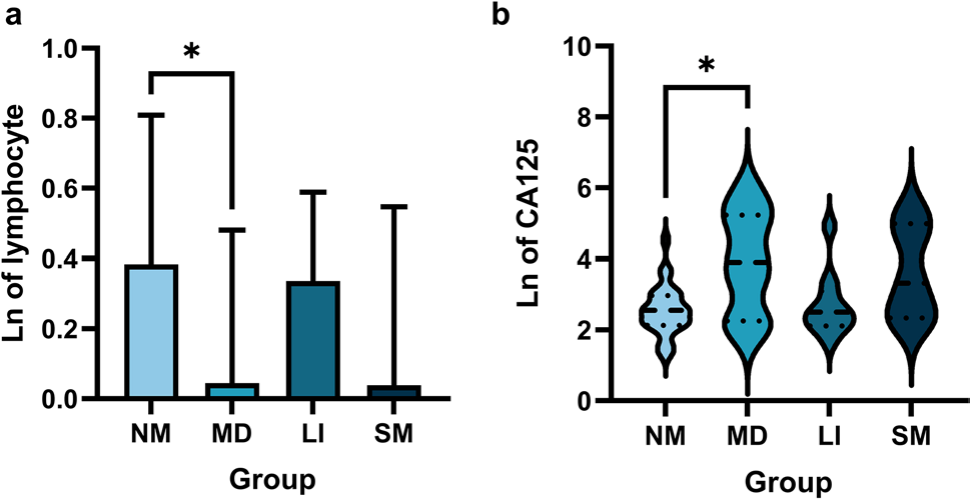
Concentration of plasma lymphocyte and serum CA125 in CRC patients according to sites of metastases. (**a**) Lymphocyte (*P* = 0.0239) were able to distinguish multiple distant metastases and non-metastases. (**b**) CA125 (*P* = 0.0195) were able to distinguish multiple distant metastases and non-metastases. *P < 0.05. Data are presented as mean ± SD or median (interquartile). *NM* Non-metastases, *MD* Multiple distant metastases e.g., liver, lung, peritoneum, omentum, mesentery, bone, ovary, adrenal gland, *Li* Liver only, *SM* Single metastasis in other parts except liver e.g., lung, bone, Abdominal, peritoneum; Ln of lymphocyte, The natural logarithm of the concentration of lymphocyte; Ln of CA125, The natural logarithm of the concentration of CA125.

Based on the expression of various indices in patients with and without metastases in the previous study, the sites of metastases were further taken into consideration, and the results showed that lymphocytes (*P* = 0.0365, Table 6, Fig. 6a) and CA125 (*P* = 0.0301, Table 6, Fig. 6b) were able to distinguish multiple distant metastases between non-metastases well. This was consistent with the aforementioned trend in the ability and expression level to distinguish between patients with and without metastasis but lacked specificity in distinguishing specific sites of metastases. The other two indices were not good at distinguishing the location of metastases, and no significant difference was found (*P* > 0.05).

Pearson’s correlation test (Table 7) was used to evaluate the correlation between the concentrations of each indicator and the clinicopathologic features of tumors. In CRC patients, EV-derived MARCKSL1 (r = 0.249, *P* = 0.028), CA125 (r = 0.391, *P* = 0.002) and lymphocyte (r =  − 0.259, *P* = 0.025) levels were significantly correlated with distant metastasis. Multivariate regression analysis was used to determine which risk factors fit the multivariate model based on univariate regression analysis (results shown as odds ratio (OR) and *P* value). Levels of EV-derived MARCKSL1 (*P* = 0.028, OR = 1.085), CA125 (*P* = 0.002, OR = 1.021) and lymphocytes (*P* = 0.025, OR = 1.021) were significantly related to an increased risk of mCRC. In the multivariate analysis, we included variables with statistical significance in the univariate logistic regression model.

**Table 7:** Associations between clinicopathological features of CRC patients and levels of tested indexes.

		Metastases	TNM	Gender	Age	MARCKSL1	CA125	lymphocyte	Lymphocyte ratio
Metastases	r	1	0.798**	0.075	0.079	0.249*	0.391**	− 0.259*	− 0.167
	*P*		0.000	0.513	0.494	0.028	0.002	0.025	0.152
TNM	r	0.798**	1	0.051	0.138	0.257*	0.299*	− 0.163	− 0.033
	*P*	0.000		0.660	0.227	0.023	0.023	0.163	0.780
Gender	r	0.075	0.051	1	− 0.037	− 0.114	− 0.050	− 0.032	− 0.064
	*P*	0.513	0.660		0.746	0.318	0.708	0.783	0.586
Age	r	0.079	0.138	− 0.037	1	0.028	0.017	− 0.053	0.050
	*P*	0.494	0.227	0.746		0.805	0.901	0.653	0.671
MARCKSL1	r	0.249*	0.257*	− 0.114	0.028	1	− 0.057	0.007	0.111
	*P*	0.028	0.023	0.318	0.805		0.672	0.950	0.342
CA125	r	0.391**	0.299*	− 0.050	0.017	− 0.057	1	− 0.544**	− 0.644**
	*P*	0.002	0.023	0.708	0.901	0.672		0.000	0.000
lymphocyte	r	− 0.259*	− 0.163	− 0.032	− 0.053	0.007	− 0.544**	1	0.693**
	*P*	0.025	0.163	0.783	0.653	0.950	0.000		0.000
Lymphocyte ratio	r	− 0.167	− 0.033	− 0.064	0.050	0.111	− 0.644**	0.693**	1
	*P*	0.152	0.780	0.586	0.671	0.342	0.000	0.000	

### Diagnostic value of MARCKSL1 in mCRC diagnosis

**Figure 7:**
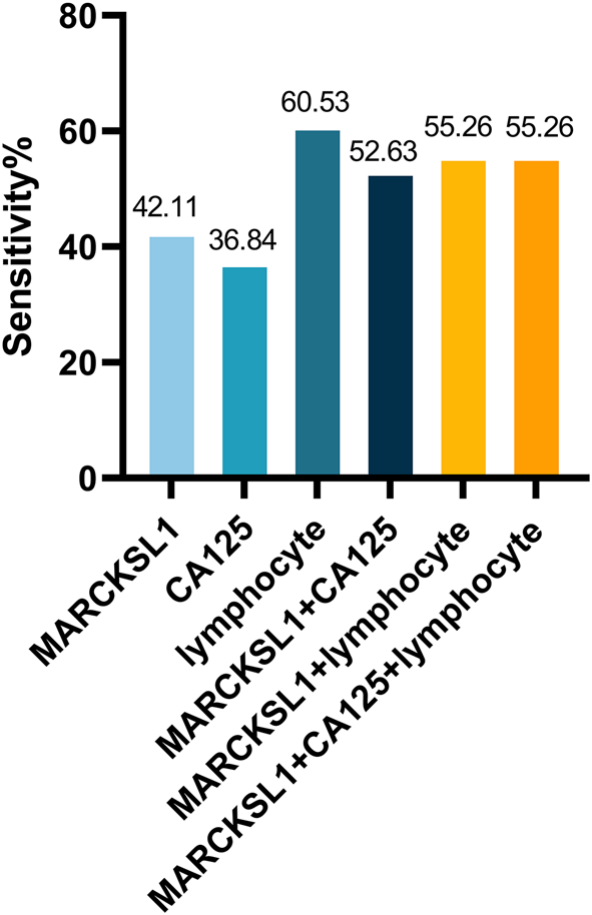
Tested indicators’ diagnostic sensitivity. The diagnostic specificity of MARCKSL1 (67.5%) was higher than that of lymphocyte (60%), but lower than that of CA125 (92.5%). MARCKSL1 combined with CA125 and lymphocyte could improve the original specificity of MARCKSL1.

**Figure 8:**
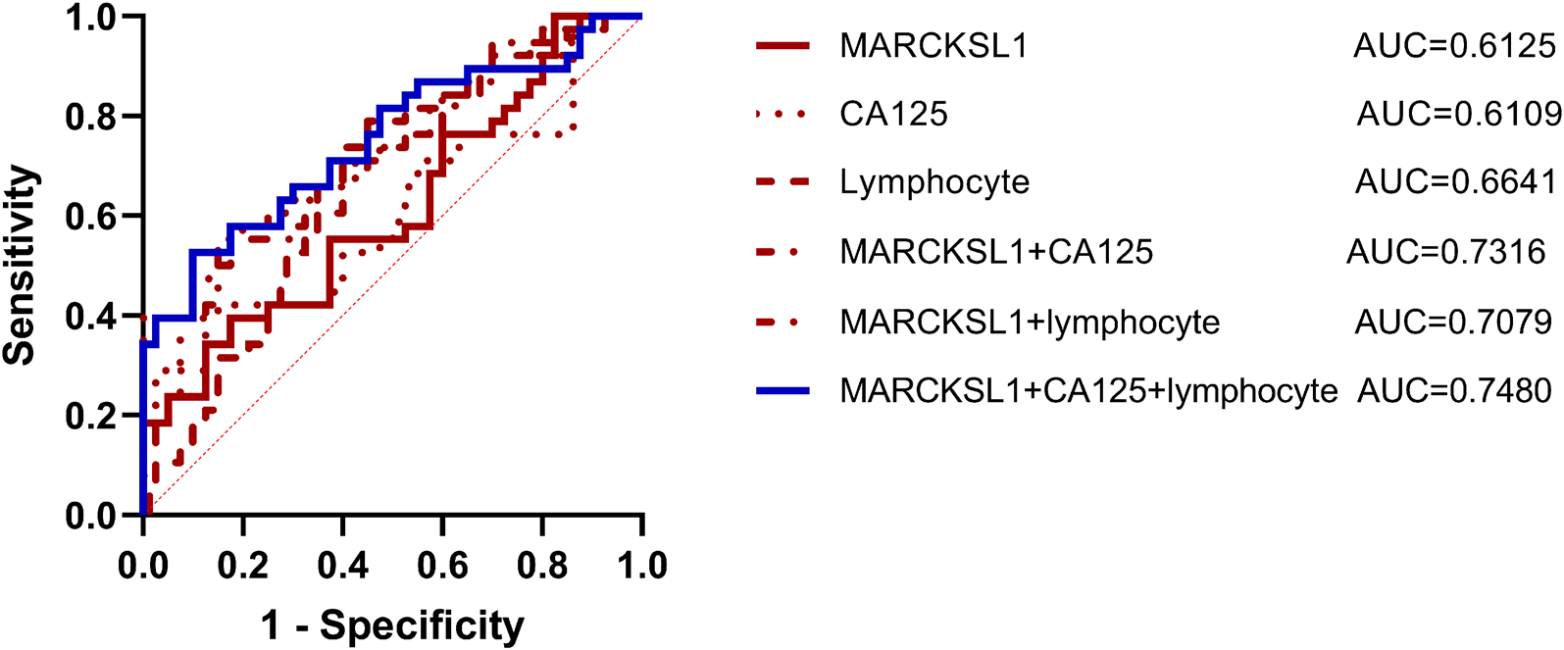
Analysis of receiver operating characteristic curves (ROC) for indicators. The area under ROC curve (AUC) of MARCKSL1 (0.6125; *P* < 0.05) was bigger than that of CA125 (0.6109; *P* < 0.05), but smaller than lymphocyte (0.6641; *P* < 0.05). MARCKSL1 combined with CA125 (0.7316; *P* < 0.05) or lymphocyte (0.7079; *P* < 0.05) were bigger than the AUC of the three indicators alone, and the combination of the three indicators could obtain the maximum AUC value (0.7480; *P* < 0.05).

To evaluate the potential value of MARKCSL1 in mCRC diagnosis, diagnostic sensitivity, specificity, accuracy, positive predictive value (PPV), negative predictive value (NPV), and area under the ROC curve (AUC) were calculated. The diagnostic sensitivity of MARCKSL1 (42.11%) was higher than that of CA125 (36.84%) and lower than that of lymphocytes (60.53%). MARCKSL1 combined with CA125 (52.63%), lymphocytes (55.26%) and the combination of all three (55.26%) could improve the diagnostic sensitivity to a certain extent, but the difference between different combinations was not significant. The sensitivity of different combinations remained lower than that of lymphocytes alone (Fig. 7). In addition, we evaluated the diagnostic specificity of the tested indicators. The diagnostic specificity of MARCKSL1 (67.5%) was higher than that of lymphocytes (60%) but lower than that of CA125 (92.5%). MARCKSL1 combined with CA125 and lymphocytes, as well as the combination of three indices (70%), could improve the original specificity of MARCKSL1. The AUC represents the clinical value of the indicator in diagnosing the disease. The AUC of MARCKSL1 (0.6125; *P* < 0.05) was larger than that of CA125 (0.6109; *P* < 0.05) but smaller than that of lymphocytes (0.6641; *P* < 0.05). MARCKSL1 combined with CA125 (0.7316; *P* < 0.05) or lymphocytes (0.7079; *P* < 0.05) was larger than the AUC of the three indicators alone, and the combination of the three indicators obtained the maximum AUC value (0.7480; *P* < 0.05), which indicated that the combination of the three indices was the most valuable in the diagnosis of mCRC (Fig. 8).

## Discussion

In recent years, we have found that the mortality and morbidity of colorectal cancer have gradually increased, and most CRC patients die due to the occurrence of distant metastases. Among these patients, only approximately 20% have had distant metastases at the first diagnosis. More than 50% of nonmetastatic CRC patients will develop distant metastases from local lesions^16^. It is very important to find convenient, noninvasive, and more sensitive markers of metastatic colorectal cancer as early as possible.

Among all body fluid tests, blood tests are widely used in clinical practice and have the advantages of being noninvasive and rapid. A variety of disease-suggestive substances are widely found in blood, among which EV-derived proteins have greater potential as markers than other substances in blood: (i) EVs contain a variety of components, among which the levels of protein, RNA and DNA can be used for cancer detection^17^; (ii) EVs are widely distributed in body fluid and can penetrate the tissue barrier, which is more convenient for clinical detection^18^; (iii) the structure of EVs can protect their contents from being degraded by enzymes in the blood circulation, and the detection results are more stable; and (iv) EVs can be purified in vitro. After purification, the concentration of markers in the blood is higher, and they are easier to detect. Therefore, EVs have higher detection sensitivity, and this characteristic is very consistent with the needs of early cancer diagnosis tools^7^.

At present, research on EV-derived protein as a diagnostic marker of colorectal cancer mainly focuses on the early diagnosis of CRC^11^, and the diagnostic ability of the mCRC diagnostic markers that have been reported is temporarily lower than that of the previous diagnostic markers related to CRC. For example, Chen et al. found that the AUC of CEA to distinguish primary CRC from mCRC was 0.65^19^. Huang et al. analyzed data from 356 patients with colorectal cancer and evaluated the diagnostic value of HSP90, circulating tumor cells (CTCs), CEA, and CA199 for distinguishing patients with colorectal cancer liver metastases and found that the AUCs of HSP90, CEA, CA199, and folate receptor (FR)-CTC + HSP90 were 0.71, 0.67, 0.60, and 0.79, respectively^20^. Compared with their study, the area under the single MARCKSL1 ROC curve in our study was slightly lower, but the combined use of CA125, lymphocytes and other indicators significantly improved the diagnostic effectiveness, and the combined indicators are commonly used clinical indicators, which have high clinical application value. Moreover, our study found that EV-derived MARCKSL1 had the ability to discriminate metastatic sites (liver), and our study covered the validation and analysis of TNM stage, lymph node metastasis status and metastatic sites. We found that EV-derived MARCKSL1 had some potential in distinguishing nmCRC from mCRC, and this topic is worth further investigation.

As a protein associated with the occurrence and development of various tumors, MARCKSL1 has been mainly verified as a potential tumor marker or a new therapeutic target in lung cancer^21–24^, liver cancer^25–27^, colorectal cancer and other tumors. In addition, there are ongoing studies on prostate cancer^28^, breast cancer^29,30^, esophageal cancer^31^, basal cell carcinoma^32^, squamous cell carcinoma^33^ and other tumors, and their potential as markers is gradually attracting the attention of researchers. MARCKSL1 has been selected as a potential marker for distinguishing metastatic and nonmetastatic sporadic colorectal cancer (sCRC) and is highly expressed in patients with metastatic sCRC. However, MARCKSL1 was not used as a final target marker for subsequent observational studies. Therefore, its specific energy efficiency as a marker of colorectal cancer has not been reported. In this study, MARCKSL1 was initially defined as a specific marker for distinguishing metastatic and nonmetastatic colorectal cancer, but it was not sensitive in distinguishing healthy and newly diagnosed nonmetastatic colorectal cancer, which was analogous to the results of previous studies^15^.

MARCKS and MARCKSL1 are closely linked to tumor development, and part of the mechanism that has been clarified is immune-related, mainly in its widespread presence in innate immune cells and its involvement in the regulation of immune function^13^. Immunotherapy is widely used in the clinical practice of treating cancer, and its basis is the tumor recognition and cell lysis activity of natural killer cells (NK cells). As the key lymphocytes of the tumor immune response, natural killer cells (NK cells) play an important role in fighting against cancer development^34^. It has been found that inflammatory factors cause a decrease in NK cell killing, while tumor cells cause an increase in NK cell killing, where the expression of several genes, including MARCKSL1, in NK cells is not affected by inflammatory factors and is only associated with exposure to tumor cells^35^.

CEA, CA19-9, CA125, CA242 and CA724, known as broad-spectrum tumor markers, have been studied and confirmed to be closely related to colorectal cancer and serve as the main indicators for the diagnosis and evaluation of colorectal cancer^36^. The most common tumor markers currently used to diagnose and evaluate patients with CRC are CEA, CA19-9, CA125 and CA242^37–39^. Compared to other markers, CEA still lacks specificity as a broad-spectrum tumor marker. CA125 has been reported as an important independent prognostic factor in CRC patients, and its diagnostic value is better than that of CEA^39^. A study confirmed that CA125-rich EVs may be markers of CRC metastasis^40^. Therefore, we expected to use CA125 as a target marker to further explore its ability to specifically differentiate metastatic colorectal cancer.

The most likely site of distant metastasis in colorectal cancer is the liver, and the prognosis of patients with liver metastasis of colorectal cancer is poor^41^. Therefore, liver metastasis was selected as an independent subgroup for analysis, and we expected that the selected indicators could be capable of distinguishing metastatic sites. However, our results demonstrated that CA125 in the plasma of patients with multiple metastases was elevated compared with patients without metastases (*P* = 0.0195), while the level of lymphocytes was higher in patients without metastases (*P* = 0.0239), both of which could only distinguish nonmetastatic colorectal cancer from colorectal cancer with multiple distant metastases to a certain extent. It is not possible to distinguish distant metastasis in different locations. MARCKSL1 and lymphocyte ratio were not associated with this subgroup analysis.

According to our results on the correlation between the concentration of each indicator and the clinicopathological features of tumors, in addition to the correlation with distant metastasis, we further found that MARCKSL1 (r = 0.257, *P* = 0.023) and CA125 (r = 0.299, *P* = 0.023) were both correlated with TNM stage to a certain extent. This result is in agreement with the findings of a previous study in which we found that MARCKSL1 was significantly different in distinguishing stage IV and stage 0-II colorectal cancer, and the expression level of MARCKSL1 in stage IV patients was significantly higher than that in stage 0-II patients (*P* = 0.0294). Although lymphocytes showed a significant difference in distinguishing between stage III and IV colorectal cancer, the expression of lymphocytes was the highest in stage III patients, significantly higher than that in stage IV patients (*P* = 0.0274) and stage 0-II patients (*P* > 0.05). The results were not ideal, which may also be related to the fact that there was no correlation with TNM stage. At the same time, we further analyzed the data of CA125 in distinguishing TNM stages, and we found that the level of CA125 in stage IV CRC patients was higher than that in stage 0-II and III patients (*P* = 0.0584), which may have certain differentiation potential. We considered that the specific differentiation ability of CA125 for stages should be further verified by expanding the sample size.

In addition to the above findings, there are still some limitations in this study. First, the number of cases included in the study is small, and the collected cases are from only one hospital, which may lead to biases in the results. Second, this study is a cross-sectional study, which only investigates the differentiation effect of EV-derived MARCKSL1 and clinical indicators on patients with nmCRC and mCRC. Further longitudinal clinical studies are needed to confirm the role of the above indicators in the clinical treatment and prognosis of CRC patients. We also plan to verify the mechanism of EV-derived MARCKSL1 in mCRC by more in vitro and in vivo experiments in the future.

## Conclusion

Circulating EV-derived MARCKSL1 is significantly elevated in the plasma of patients with metastatic colorectal cancer and can be used as a potential diagnostic marker for the early diagnosis of mCRC. Furthermore, it can be combined with other diagnostic markers or indicators to improve diagnostic efficacy in clinical practice. The mechanism of MARCKSL1 in colorectal cancer metastasis should be further studied to guide clinical treatment.

## Supplementary Information

Supplementary Information.

## Data Availability

The datasets generated during and analysed during the current study are available from the corresponding author on reasonable request.
