# Supplementary material for: Circulating extracellular vesicle-derived MARCKSL1 is a potential diagnostic non-invasive biomarker in metastatic colorectal cancer patients
Source: Sci Rep. 2023 Jun 20;13:9957. doi: 10.1038/s41598-023-37008-0 (PMC10281964; doi:10.1038/s41598-023-37008-0)
Supplement: Supplementary file 1 — Supplementary Information. [file 41598_2023_37008_MOESM1_ESM.pdf]

Supplementary Information

Circulating extracellular vesicle-derived MARCKSL1 is a potential diagnostic non-invasive biomarker in metastatic colorectal cancer patients

Author: Wenqing Rong[1], Shiyun Shao[1], Yunzhou Pu[1], Qing Ji[1] and Huirong Zhu[1]

[1]Department of Medical Oncology, Shuguang Hospital, Shanghai University of Traditional Chinese Medicine, Shanghai 201203, China

Correspondence: Qing Ji, E-mail: ttt99118@hotmail.com

Huirong Zhu, E-mail: huirong_z@163.com

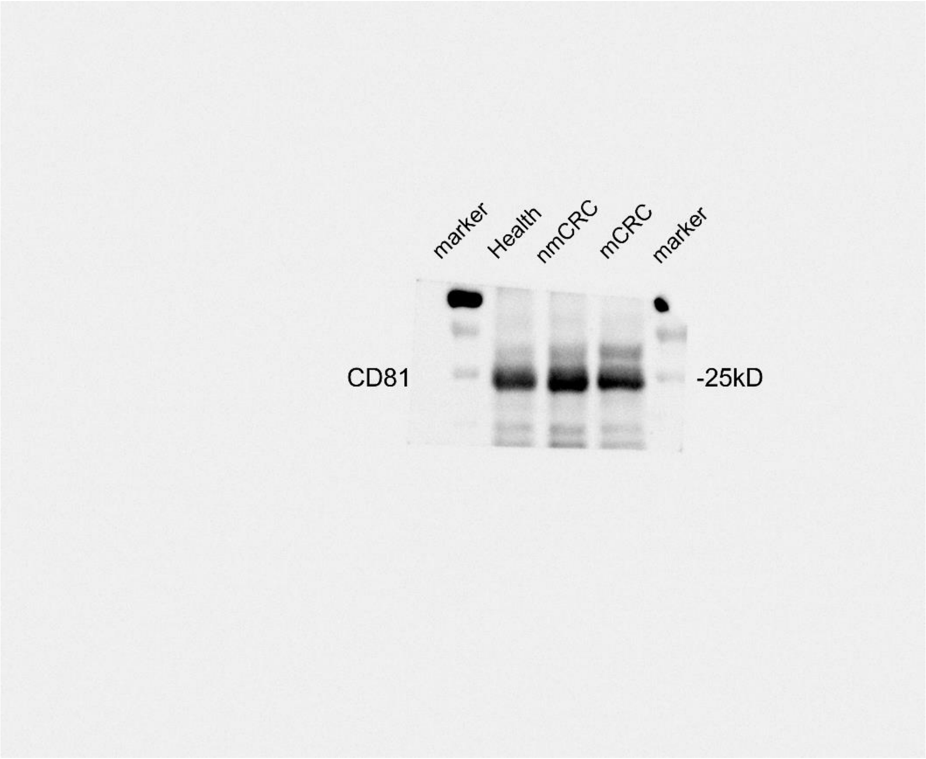

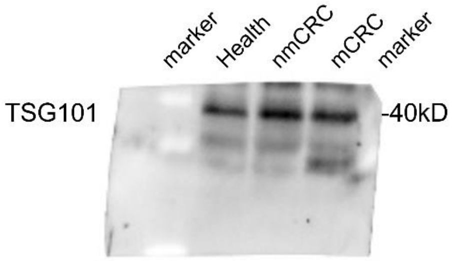

Supplementary Figure 1. Original Western blot blots of Figure 1c

Western blots were cropped prior to incubation with primary antibody hybridization.
